# Ocular Surface Parameters in Glaucoma Patients Treated with Topical Prostaglandin Analogs and the Importance of Switching to Preservative-Free Eye Drops—A Systematic Review

**DOI:** 10.3390/life15121837

**Published:** 2025-11-29

**Authors:** Jaromir Wasyluk, Grzegorz Rotuski, Marta Dubisz, Radosław Różycki

**Affiliations:** 1 Military Institute of Aviation Medicine, 01-755 Warsaw, Poland; 2 OPTIMUM Bracka 11/13, Medical Center in Warsaw, 00-501 Warsaw, Poland; 3 ORBITA Mińska 25a/lok.U10, Medical Center in Warsaw, 03-808 Warsaw, Poland

**Keywords:** preservatives, ocular surface disease, osmometry, interferometry, bulbar redness, ophthalmic imaging, diagnosis of eye diseases

## Abstract

**Background:** The use of preservative agents in eye drop solutions may worsen symptoms of ocular surface disease, which is a highly prevalent syndrome worldwide. Preservatives are often used in pharmacotherapy of glaucoma, another disease concerning tens of millions of people around the globe. These numbers are predicted by the World Health Organization and are predicted to increase with time due to constant aging of populations. **Methods:** PubMed and Scopus databases were searched for articles investigating the topic of ocular surface disease in relation with glaucoma pharmacotherapy, according to Preferred Reporting Items for Systematic Reviews and Meta-Analyses guidelines. The aim of this review is to summarize the effect of various solvents used in drug formulations and ways to quantify their impact on the ocular surface. **Discussion and Conclusions:** Topical ophthalmic preservative-free formulations are better tolerated and less burdensome for all patients. They should be considered especially for glaucoma patients, who are expected to take medications for years, up to decades or a lifetime in many cases. Due to the chronicity of dry eye disease and the lack of reliable ways for lacrimal and meibomian gland renewal, primary prophylaxis is of uttermost importance. Unfortunately, despite the development of many measuring devices, the standardization of diagnostic methods poses a challenge due to high variability of results which are influenced by a myriad of factors—local, internal, and external.

## 1. Introduction

Glaucoma is a chronic progressive optic neuropathy, leading to visual field deterioration. This is the first cause of irreversible blindness globally and the second cause in developed countries (after age-related macular degeneration). According to the European Glaucoma Society as well as the guidelines of ophthalmological national regulators, prostaglandin topical monotherapy is the first-line therapy in most cases of primary open glaucoma (POAG), the most common type of the disease [1].

Prostaglandin analogs (PGAs) are among the most commonly used classes of medications in the treatment of POAG and elevated intraocular pressure. They reduce intraocular pressure (IOP) primarily by increasing the uveoscleral outflow of aqueous humor. In ophthalmic practice, the following four prostaglandin analogs may be used: latanoprost, travoprost, bimatoprost, and tafluprost. Latanoprost is the most commonly prescribed PGF_2_α analog, with proven efficacy and a favorable safety profile [2]. Travoprost is similar in efficacy to latanoprost (both reduce intraocular pressure by 25–32%), with some differences in tolerability depending on the formulation [3]. Bimatoprost may have a slightly greater IOP-lowering effect (up to 40%) but is also associated with a higher incidence of side effects [4]. Tafluprost is a newer agent available in preservative-free formulations, shown to be effective and better tolerated by some patients with ocular surface sensitivity [5,6]. Topically applied PGAs in eye drops are generally well-tolerated, but local side effects may sometimes be clinically relevant. They can be broadly classified into those involving the ocular surface and its associated protective apparatus as well as those affecting the anterior and posterior segments of the eye [7].

**Figure 1:**
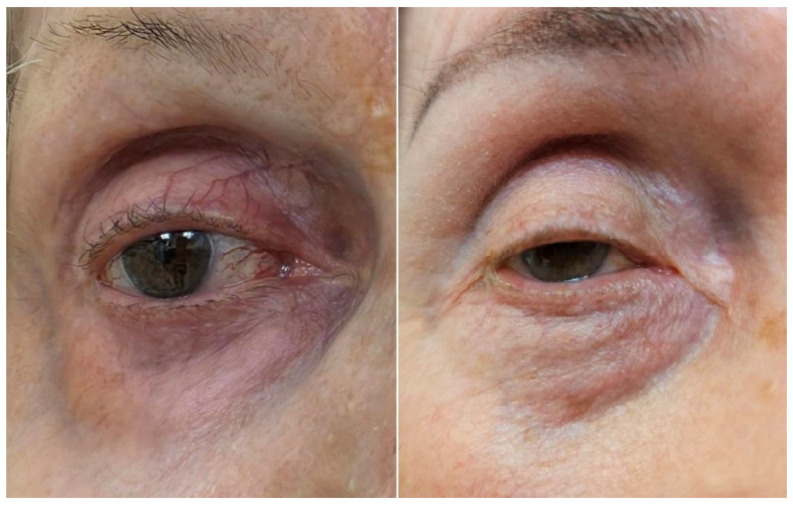
Prostaglandin-associated periorbitopathy.

The first group includes mild conjunctival hyperemia, which is frequently observed especially with bimatoprost and travoprost [4], darkening of the periocular skin and eyelashes after long-term use, hypertrichosis, trichiasis, and distichiasis—eyelashes abnormalities typically become apparent after several weeks or months of treatment. It appears that the side effects mentioned are reversible upon discontinuation of treatment [2,8]. Ocular irritation or dryness usually occurs but tends to be mild and transient [9]. The use of PGA eye drops can also lead to the development of prostaglandin-associated periorbitopathy. This syndrome involves changes in the orbital area, including deepening of the upper eyelid fold, ptosis of the upper eyelid, enophthalmos, and exposure of the scleral band above the lower eyelid. These symptoms are most likely the result of atrophy of the orbital fat tissue [10] in long-term use of these drops. We present these features in two cases from our clinical practice in Figure 1.

The second group of PGA side effects includes increased iris pigmentation, which is permanent and occurs mostly in patients with mixed-color irises [2,8]; iris cysts and ciliary body cysts; herpetic keratitis and anterior uveitis, which are rare adverse effects and may represent a reactivation of persistent disease in susceptible individuals [10]; cystoid macular edema, which is more common in pseudophakic patients or those who have undergone eye surgery complicated with posterior capsule disruption [11]; decrease in the central corneal thickness of unknown mechanism. A matter worth noting is the contradictory set of reports regarding melanoma in five patients occurring during the first year since treatment initiation [12]. This may very well be a coincidental finding, and more studies in oncology centers concerning the number of patients with melanoma taking PGAs matched against an equally wide population in other ophthalmic centers could help provide a solution. 

Due to some degree of overlap between the side effects of PGA and preservatives, awareness of the differences can help in deciding whether switching to a preservative-free (PF) analog or completely changing the anti-glaucomatous drug group is necessary. To the best of our knowledge, there were no trials comparing the incidence of adverse effects in patients using PGAs with preservatives and then switched to PF against patients using PF-PGAs for years and then discontinued.

In any case, early OSD onset after initiation should imply the consideration to discontinue the drug with watchful monitoring of symptoms improvement in the next few days and returning to treatment with a PF alternative. Some practitioners opt for a short course of weak steroids (e.g., 0.335% hydrocortisone) 2–3 × daily for 2–3 weeks to minimize irritation of the ocular surface, with additional benefits to meibomian gland function and inflammation mitigation and accelerating symptoms alleviation. Severe but rare sight-threatening symptoms, such as uveitis, macular edema, herpetic keratitis, or bullous keratopathy may require peribulbar steroid injections, anti-VEGF intraocular injections, or antiviral topical medications. These serious complications should be a contraindication to continuing PGA therapy.

In recent years, the topical impact of ophthalmic glaucoma drops on eye surface in persistent therapy is being widely discussed, and the need to avoid some preservatives (e.g., benzalkonium chloride—BAK) is being underlined, due to inducing or aggravating ocular surface disease (OSD). This entity, commonly called “dry eye syndrome” to facilitate patients’ comprehension, is estimated to be present in around 1/3 of the global population [13]. The morbidity is higher in urbanized and polluted areas, while the incidence increases even in children, with younger individuals being diagnosed every year [14]. Meibomian gland dysfunction (MGD) seems to be the main cause—linked with aging, increased workload using the computer (digital eye strain), post-menopausal hormonal changes, contact lens wear, and the increased incidence of autoimmune disorders [15]. Because of the irreversible meibomian gland damage, at current knowledge, primary prophylaxis should be stressed. OSD decreases visual acuity and the local discomfort transmits to overall discontent; it also impairs healing after ophthalmic medical and surgical interventions. On top of that, it requires taking lubricating eye drops, gels, and pommades chronically several times daily—the same goes for glaucoma, where pharmacotherapy requires repeating installations at specific hours every day. Because of this frequency, bottles that are supposed to last for months after opening often contain preservatives and additives for longer viability and stability, restraining the growth of pathogens. Problems caused by preservatives may result in deterioration of long-term patient compliance and persistence, causing treatment interruption or irregularity, eventually influencing the progression of glaucoma and worsening the effects of treatment in terms of protecting the visual field.

The objectives of this study are to summarize the additives contained in prostaglandin drug formulations and review their impact on the ocular and periocular regions, which poses a challenge to be adequately quantified, with many modalities coming in handy, but each having their downsides.

## 2. Materials and Methods

Following Preferred Reporting Items for Systematic Reviews and Meta-Analyses (PRISMA) guidelines [16], a systematic review was conducted. PubMed, Cochrane Library, and ClinicalTrials.gov electronic databases were searched for articles that investigated the impact of preservatives on the ocular surface using various combinations of keywords including glaucoma, prostaglandins, ocular surface disease, dry eye disease, dry eye syndrome, preservatives, additives, excipients, buffers, chelators, stabilizers, bulbar redness, inflammation, tear film. We did not restrict the research to specific timeframes. Our study has been registered in PROSPERO (ID: 1184533).

Initially we followed the PICO algorithm:

- Population—glaucoma patients;
- Intervention—pharmacotherapy with PGA eye drops;
- Comparison—correlation of ocular surface symptoms with specific substances included in eye drop formulations;
- Outcomes—quantification of adverse effects through specific diagnostic methods.

Two authors (G.R., M.D.) independently performed the database search. All the resulting titles were screened, and if suitable, the abstract was accessed. The matching topics were then inspected for full-text availability online. Disagreements were debated, and in the end, the final decision was made by the main author (J.W.). Similarly, the risk of bias was evaluated according to the guidelines of the Cochrane Handbook for Systematic Reviews of Interventions by two authors (G.R., M.D.) independently, and disagreements were solved again by another author (R.R.). The Grading of Recommendations Assessment, Development, and Evaluation (GRADE) was used for certainty evidencing.

**Figure 2:**
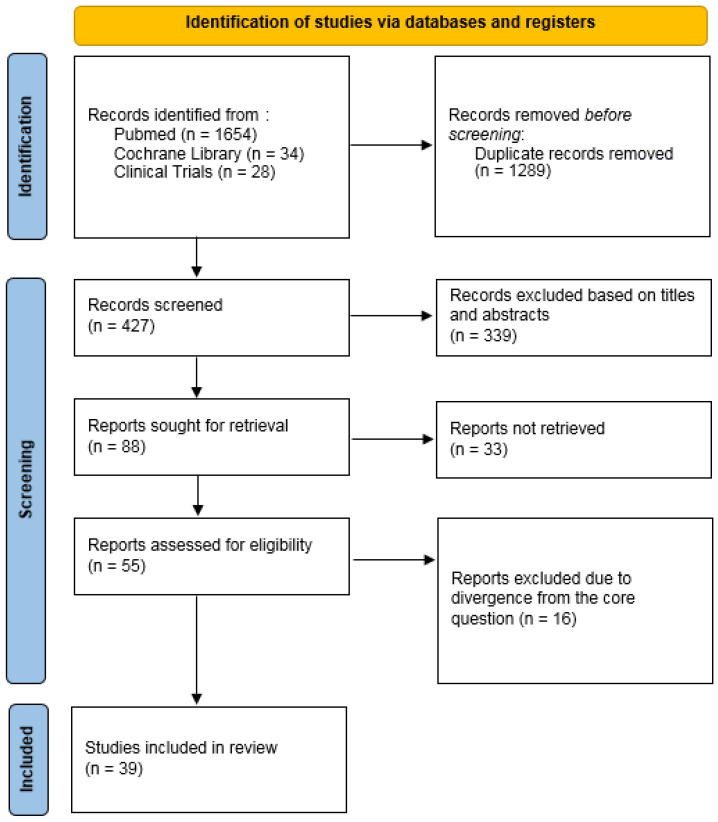
Flow chart of the literature search.

In total, 1716 articles were identified, of which 1289 duplicates were removed before screening. Randomized controlled trials (RCTs), cohort, case–control, and cross-sectional studies were prioritized over case reports, narrative reviews, editorials, and in vitro studies. Further reviewing led to rejecting articles lacking full-text accessibility, unavailable in English, and studies performed on animals. Out of the 88 reports retrieved, 33 were excluded and the remaining 55 were eligible, narrowing them further by coherence with the subject in question. Therefore, in total, 39 articles were included in this review (Figure 2).

## 3. Results

After this analysis, we summarized the preservatives and excipients used in glaucoma eye drop formulations and their impact on the ocular surface. BAK is by far the most prevalent preservative component in ophthalmic drops [17]. Other classes of alternative preservatives in glaucoma eye drops include polyquaternium-1 (Polyquad), which acts as a detergent; stabilized oxychloro complex (SOC; Purite), an oxidizing agent; and SofZia, an ion-buffered preservative [18]. Excipients, which are pharmacologically inactive substances serving as carriers for active ingredients, can also affect the ocular surface [19].

The majority of studies focus on BAK, as it is the most widely used and is considered the most toxic preservative. Other preservatives have demonstrated lower levels of toxicity, are better tolerated, and are considered safer for long-term use. Typically, there is an inverse relationship between a preservative’s antimicrobial effectiveness and its tolerance by the ocular surface [20].

We did not find robust studies concerning the reversal of OSD changes upon PGA eye drop withdrawal, discerning formulations with and without preservatives. This seems to be a field worth further investigation. Therefore, the most commonly used preservatives and excipients used in glaucoma eye drops and their adverse effects are presented in Table 1. 

## 4. Discussion

Preservatives themselves may induce or aggravate already present OSD in some patients, provoking several symptoms such as irritation, redness, tearing, foreign body sensation, punctate epitheliopathy, etc. [21]. Their primary purpose is to impede microbial growth in water-based drug formulations. BAK is one of the most commonly used preservatives in all types of eye drops. Aside from keeping the solution sterile, it acts as a surfactant reducing surface tension and, thus, allowing better penetration of the medication across the entire ocular surface. Adverse effects attributed to BAK, including conjunctival inflammation and fibrosis, tear film instability, corneal cytotoxicity across all its layers, anterior chamber inflammation, trabecular meshwork cell apoptosis, cataract development, macular edema, and even systemic effects, have been well documented [22]. These effects can lead to ocular discomfort, poor IOP control, glaucoma surgery failure, and decreased patient compliance. The influence of BAK has been already widely studied, causing its subsequent withdrawal from many drug formulations in the markets. Several pharmaceutics companies implemented micro-bottles containing a single dose of the drug, avoiding the need for preservatives. However, due to increased packaging complexity, costs increase and more plastic waste is produced, raising another limitation. 

Preservatives can damage all glands and cells responsible for the production and stability of the tear film, as proven by multiple studies [23], and any sign of deficiency should imply higher caution towards ophthalmic drugs administered by the patients regarding preserving ocular surface integrity. For instance, the cytotoxic effect of BAK is intensified under hyperosmotic conditions. Since BAK is known to disrupt the tear film, which may contribute to the development of evaporative dry eye and tear hyperosmolarity, it can create conditions that further amplify its own cytotoxicity. In an in vitro model, Clouzeau et al. highlighted the risk of initiating a vicious cycle and emphasized the need to avoid BAK, particularly in patients with dry eye syndrome [24]. Recently, attention has also been drawn to the potential adverse effects on the ocular surface caused by eye drops containing phosphate buffers, especially in cases of corneal epithelium damage or prolonged use of eye drops containing these compounds. Phosphates are well tolerated by a healthy ocular surface. However, problems arise when they are used in patients with a damaged corneal barrier, such as in cases of dry eye syndrome, injuries, or inflammatory processes. The European Medicines Agency (EMA) has highlighted the risk of developing superficial band keratopathy and deeper corneal calcifications as a result of using phosphate buffers in eye drops [25].

### 4.1. Phosphates

The role of phosphates in eye drops is to maintain the correct pH of the solution (buffer system). They increase the solution’s stability, facilitate the dissolution of certain active substances, and improve the comfort of using eye drops; therefore, they are widely used due to their components. Phosphate can bind to calcium in eye fluids and may cause the formation of deposits of calcium phosphate; that is why there was concern that they can cause corneal calcification. The risk factors that may contribute to the formation of calcium phosphate deposits include increased osmolarity of the tear film caused by excessive evaporation, elevated pH resulting from corneal metabolism, increased levels of calcium or phosphates, and the presence of preservatives such as BAK, which enhances the toxicity of phosphates by damaging the corneal epithelium [26]. The German Medicines Regulatory Agency was the first to draw attention to this problem. As phosphates are widely used in many countries, the Committee for Medicinal Products for Human Use (CHMP) conducted an evaluation based on data collected from various parts of Europe. After reviewing the reported cases of corneal calcification in patients using phosphate-containing eye drops, the CHMP concluded that these adverse effects were very rare. At the same time, there were also many cases of corneal calcification in patients who had not used such drops. Based on this, the CHMP determined that the benefits of using phosphate-buffered eye drops outweigh the risks and that they can continue to be used in the EU. However, to ensure that both prescribers and patients are informed about the issue, it was advised that the product information for these medicines should be updated [27].

### 4.2. Slit-Lamp Assessment

With new modalities of OSD treatment emerging, thorough diagnostics are necessary. All tear film layers are important to protect and equally nourish the totality of the corneal surface that receives oxygen and nutrients only from the exterior. Imbalance causes disruption in the continuum of the tear film, leaving some areas vulnerable. Hence, it is important to distinguish which of the layers may be the culprit behind OSD. Schirmer test is one of the basic methods of testing aqueous-deficient dry eye disease (DED), available in most ophthalmology settings [28]. Lissamine green stains epithelial cells unprotected by the mucous layer, showing also the state of the bulbar conjunctiva, which cannot be visualized with standard fluoresceine test [29]. Meibography is an examination of the meibomian glands upon reversal of the eyelid margin—infrared exposes their course beneath the palpebral conjunctiva. Their state is crucial to providing a lipid layer, preventing tears from evaporating. Shortening of the gland length reveals atrophy that is usually due to clogging of the vesicles and inability to secrete meibum from the orifices that should be present just in front of the Marx line, which delimits the area between the conjunctiva and squamous epithelium of the skin [30]. This feature should be examined along with the consistency of the meibum upon squeezing the eyelid margin—this can be performed with atraumatic forceps or with a Maskin probe; however, the latter can cause scarring, leading to meibomian gland progressive dysfunction in the long run [31]. Issues with visualizing the totality of these glands concern patients with tight eyelids—occurring with senescence, especially in predisposed individuals, due to local fibrosis of adjacent structures [32]; but this process is accelerated by adiposal tissue atrophy [33], related to instillation of prostaglandins over the years. Therefore, the extent of meibomian gland atrophy may be hard to assess repeatably.

### 4.3. Quantification of OSD with Scales

**Figure 3:**
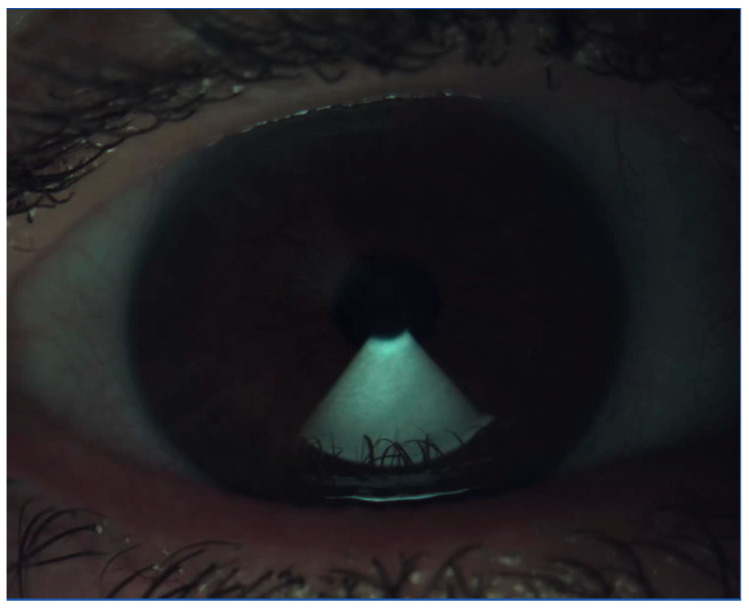
Interferometry measurement. IDRA Ocular Surface Analyzer (SBM Sistemi, Rivalta di Torino, Italy).

To examine the condition of the ocular surface, various diagnostic methods and multi-modal imaging should be integrated. For instance, interferometry (Figure 3), measuring the thickness of the tear film, can be used to monitor the clinical response after each treatment session in time. It can also play an important role in assessing if ocular biometry performed for IOL calculation is reliable, since the corneal curvature is evaluated along with the tear film, which ensures the optical quality of the cornea’s refractive power by providing a smooth refracting surface, hence, influencing the results of the implant power chosen for the patient [34]. Variable results in patients on follow-up visits may be due to the multitude of factors influencing this parameter, including the vast range considered as a normal value (40–100 nm) [35]. The excretion of meibum depends on the quality of blinking [36] and may also be related to dietary habits, with a higher consumption of omega-3 fatty acids improving meibum consistency. LIPCOF (lid-parallel conjunctival folds) scale accounts for the number of conjunctival folds, assessed above the lower eyelid margin border and is proportional to the degree of OSD [37]. This needs to be differentiated with conjunctival laxity, observed in the elderly, but both can disturb the stability of the tear film due to an unequal surface upon blinking.

**Figure 4:**
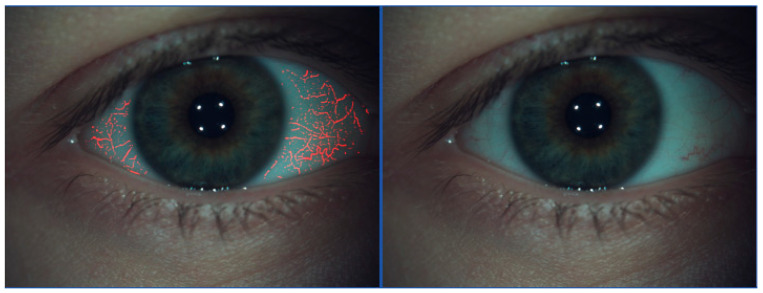
Bulbar redness assessment. IDRA Ocular Surface Analyzer (SBM Sistemi, Italy).

The McMonnies scale indicates the degree of bulbar redness (Figure 4). A known effect of topical prostaglandins is conjunctival hyperemia, resulting from the mechanism of action of enhancing suprauveal aqueous humor outflow [38]. This class of drugs allows for the highest amount of IOP reduction compared to other solo medications.

### 4.4. Tear Film Osmolarity

Tear film osmolarity reflects the balance between tears production and evaporation. It is a relatively simple and fast diagnostic test, consisting of sampling the tear meniscus by gently touching the eye surface in proximity of the eyelid margin. Higher osmolarity levels are positively correlated with the severity of ocular discomfort [35]. According to the TFOS DEWS III (Third Tear Film and Ocular Surface Society Dry Eye Workshop) criteria, a value of ≥308 mOsm/L in either eye or an interocular difference of >8 mOsm/L is an objective measure to assess for OSD, measured with the TearLab device [39]. Testing osmolarity of the tear film allows for better differentiation between evaporative DED and aqueous deficiency, which can hint on the best therapeutic strategy for the patient and can be a useful tool in tracking the effectiveness of medical interventions overtime. However, the degree of variability and the impact of multiple internal and external factors on the composition of tears are hindering the reliability of osmometry as a standalone test [40,41]. For instance, diabetic patients have weakened corneal sensitivity as a result of polyneuropathy occurring with prolonged hyperglycemia, which translates to epithelial defects, inducing inflammation of the ocular surface and hyperosmolarity of the tear film [42]. Changes can be demonstrated with contact and non-invasive esthesiometers [43] and should be performed in at least four different peripheral quadrants of the cornea along with the center. Again, hypoesthesia can be derived from a multitude of possible causes, ranging from Herpes infection to various neurological diseases [44]. Osmolarity is also influenced by patients’ everyday environment—polluted city centers, self-hygiene, dietary habits, air humidity, and temperature [45,46]. As shown in trials, it quickly goes back to baseline after instillation of lubricating eye drops [47] but decreases and settles when inflammation is being treated, e.g., with steroids or cyclosporine [48]. This proves the importance of prescribing solely preservative-free glaucoma eye drops, since inducing inflammation leads to a chain of destructive events, which are hard to mitigate or reverse with simple lubrication.

### 4.5. Other OSD Evaluation Modalities

**Figure 5:**
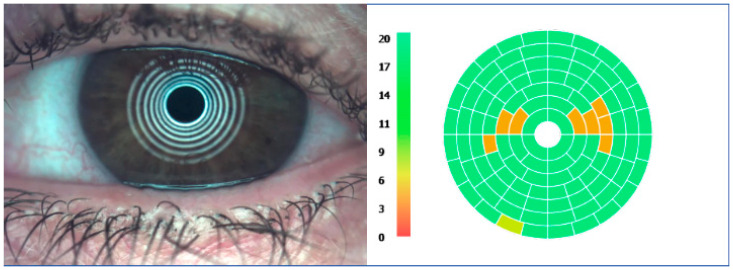
NITBUT measurement. IDRA Ocular Surface Analyzer (SBM Sistemi, Italy).

Another interesting diagnostic method is thermography of the eyelids and ocular surface. Performed with an infrared camera detecting the heat emitted from particular tissues, it converts this data into a map with colors designating specific temperature brackets. Thermography helps evaluate non-invasive tear break-up time (NITBUT, Figure 5) through thermal patterns [49,50]. Local heat spots on the lid margin or conjunctiva may correlate with inflammatory activity. This test reflects tear film thickness and lipid integrity and can help differentiate between evaporative and aqueous-deficient subtypes of DED: in the first category, a rapid temperature drop after blinking occurs due to lipid layer deficiency; in the other baseline, temperatures may be lower with a slower recovery after blinking. Thermography can also be used to assess the efficacy of interventions like warm compresses, anti-inflammatories, and meibomian gland therapy (thermal pulsation, intense pulsed light) [51,52]. The downsides to this test encompass no established diagnostic standards, and interpretation depends on experience analyzing the software, which requires frequent calibrations due to changing temperatures and humidity in a typical examination room. External sources of light can also disturb temperature readings.

The psychological part of the assessment is equally relevant, since OSD can have a negative impact on activity and psychomotor drive [53]. Moreover, chronic systemic illnesses can cause depression, exacerbating ocular surface inflammation and dryness, which may be further induced by anti-depressants such as SSRI. For these reasons, a holistic approach for the patient, even in an ophthalmology clinic, is necessary in reluctant cases, and awareness of detailed medical history should be an inherent part of the visit.

Limitations of this study include the sole analysis of prostaglandins with and without preservatives on the ocular surface. Comparing these findings with other drug groups used in the pharmacotherapy of glaucoma would expose changes resulting from the side effects of preservatives alone.

## 5. Conclusions

Preservative-free latanoprost (notably without BAK and phosphates) is a well-tolerated drug formulation that should be considered for all glaucoma patients requiring pharmacotherapy as a first-line treatment. Switching glaucoma eye drops to preservative-free and additive-free analogs should be considered at every ophthalmic visit to preserve tear film stability and to improve OSDI as well as the overall well-being of patients. Worsening of ocular surface parameters in glaucoma patients is expected due to involutional changes related to aging, increased autoimmunity, and poor blinking, probably related to continuous gaze focus during screen time. The individual changes can be measured through multi-modal imaging methods and the results displayed to patients in order to improve patient compliance with the tailored treatment. As for the exact cause of OSD onset, finding the primary underlying mechanism of the vicious cycle through cost-efficient methods and monitoring the effects in time through objective credible methods still pose a challenge nowadays and warrant further research. Possibly, integrating a careful selection of results into scientific models aided by artificial intelligence could help make a step forward in this aspect. As stated above, more studies are needed to quantify the effect of eye drop withdrawal—either PGAs vs. no glaucoma eye drops, switching to preservative-free analogs, a comparison of OSD changes reversal in correlation with the time they were used, and the influence of pre-existing OSD prior to treatment initiation. This would further validate the importance of balancing the risk–benefit ratio of glaucoma pharmacotherapy in different configurations.

## Figures and Tables

**Table 1:** Preservatives and excipients used in glaucoma eye drops and their adverse effects.

Substance Name	Type	Purpose in Formulation	Potential Adverse Effects	Treatment Options
Benzalkonium chloride (BAK)	Preservative	Antimicrobial—dissolves cell walls and membranes	- Corneal and conjunctival epithelial toxicity - Meibomian gland dysfunction - Tear film instability - Conjunctival inflammation - Trabecular meshwork cell apoptosis - Increased risk of surgical failure	- Drug withdrawal or its replacement with preservative-free formulation - Introduction of preservative-free lubricating eye drops - Introduction of weak low-dose topical steroids (e.g., 3.35 mg/mL hydrocortisone solution) - Introduction of topical antiallergic medications (e.g., 0.25 mg/mL ketotifen solution, 1 mg/mL olopatadine solution or 20 mg/mL sodium cromoglycate solution)
Cetrimonium bromide	Preservative	Antimicrobial detergent	- Side effects similar to BAK	
Polyquad^®^ (Polyquaternium-1)	Preservative	Antimicrobial—acts on cell membranes	- Generally better tolerated - Mild irritation in sensitive individuals	
Purite^®^ (Stabilized oxychloro complex of chlorite, chlorate andchlorine dioxide)	Oxidative	Oxidation of intracellular lipids and glutathione	- Well-tolerated - Rare cases of ocular surface irritation	
SofZia^®^ (Borate, sorbitol, propylene glycol and zinc)	Ion-buffered preservative	Maintains sterility	- Minimal ocular surface toxicity - Considered safer for long-term use	
Phosphate buffers	Excipient (buffer system)	pH regulation, solubility enhancement	- Risk of corneal calcification in compromised eyes (e.g., dry eye, epithelial defects) - Band keratopathy	
EDTA (Ethylenediaminetetraacetic acid)	Chelator, stabilizer	Enhances preservative efficacy	- Possible epithelial irritation - May increase corneal permeability	
Propylparaben	Preservative	Antimicrobial agent	- Allergic conjunctivitis - Lipid layer destabilization	
Chlorobutanol	Preservative	Antimicrobial agent	- Allergic reactions - Impaired corneal epithelialization	
Sodium perborate	Oxidative	Hydrolyzed into hydrogen peroxide and borate	- Allergic reactions	
Octoxynol 40	Stabilizer	Antimicrobial—cell lysis and membrane permeabilization	- Eye redness and irritation	
Chlorhexidine	Antiseptic	Bacteriostatic	- Allergic reactions	
Glycerol, Hyaluronic acid, PEG/PPG	Humectants, excipients	Moisturizing, improve drop viscosity and comfort	- Generally well-tolerated - Rare allergic reactions	

## Data Availability

The data used in the current study is available from the corresponding author upon reasonable request.

## Abbreviations

The following abbreviations are used in this manuscript:

DED	Dry Eye Disease
OSD	Ocular Surface Disease
OSDI	Ocular Surface Disease Index
PF	Preservative-Free
NITBUT	Non-invasive Tear Break-up Time
LIPCOF	Lid-Parallel Conjunctival Fold
RCT	Randomized Clinical Trial
